# Understanding the Prevalence Rates of Interpersonal Violence Experienced by Young French-Speaking Swiss Athletes

**DOI:** 10.3389/fpsyg.2021.726635

**Published:** 2021-12-20

**Authors:** Élise Marsollier, Denis Hauw, Fabienne Crettaz von Roten

**Affiliations:** Institut des Sciences du Sport, Faculté des Sciences Sociales et Politiques, Université de Lausanne, Lausanne, Switzerland

**Keywords:** youth, research instrument, actual experience, abuse, harassment

## Abstract

Facing the important methodological limitations of the instruments used for assessing the prevalence of interpersonal violence faced by young athletes, the aim of the present study was to propose and describe the use of a research instrument adapted to young and French-speaking athletes. In addition, by collecting preliminary data with a Swiss sample, we aimed to measure the different forms of interpersonal violence young athletes have experienced at least once during their sport practice. Our questionnaire was based on three existing questionnaires and adapted for a young audience. Regarding prevalence, results showed that among the 210 respondents, 75% declared psychological violence, 53% physical violence, 28% sexual violence and 21% reported no violence. The other results showed that this instrument appears to be well-structured to measure interpersonal violence and understandable for young athletes. Based on the strengths and limits of our instrument, the methodological need of standardization of research instruments is discussed in line with a need of more studies to fully understand the phenomenon.

## Introduction

Interpersonal violence (IV) against young athletes has recently been defined as violence between individuals including violence by an adult in a position of authority, such as the coach, violence between athletes, and violence by spectators (Parent and Fortier, 2018). While sexual violence against young athletes is one of the more concerning threats in the media (see the scandals of Dr. Larry Nassar, who sexually abused hundreds of young American gymnasts, and Sarah Abitbol, French skater who was sexually abused by her coach during her early career), other forms of IV have also been identified, such as psychological violence (e.g., Stirling and Kerr, 2013), physical violence (e.g., Stafford et al., 2013), and neglect (Willson et al., 2021). Psychological violence includes restriction of movement and patterns of belittling, denigrating, scapegoating, threatening, scaring, discriminating, ridiculing, and other non-physical forms of hostile treatment or rejection (World Health Organization, 1999). Physical violence concerns any actions of a physical nature that compromise or threaten the integrity or the physical or psychological wellbeing of a person (Clément and Dufour, 2009). Sexual violence is defined as “A sexual act that is committed or attempted by another person without freely given consent of the victim or against someone who is unable to consent or refuse” (Basile et al., 2014, p. 11). Last, neglect is described as “The failure to provide for the development of the child […] in the context of resources reasonably available to the family or caretakers and causes or has a high probability of causing harm to the child’s health or physical, mental, spiritual, moral or social development” (World Health Organization, 1999, p. 15).

The few prevalence studies in Europe and Canada showed a high percentage of occurrence of neglect as well as psychological, physical, and sexual violence. In Belgium and Netherlands, 38% of the 4,043 young sport participants retrospectively surveyed by Vertommen et al. (2016) reported at least one experience of psychological violence, 11% reported physical violence, and 14% reported sexual violence. Using the same survey (IViS; Vertommen et al., 2016) with elite sport participants in Netherlands, Belgium, and Germany, Ohlert et al. (2020) obtained different numbers: 72% reported psychological violence, 25% physical violence, and 30% sexual violence among the 1,665 elite athletes surveyed. Thus, in similar countries and using the same survey, psychological violence seems to be the most reported by former athletes, followed by physical violence and sexual violence, but the prevalence rates seem higher among former elite athletes. In the same line and searching for the potential effect of types of sport as well as levels of practice, Hauw et al. (2021) conducted the first prevalence study on IV against young athletes in French-speaking Switzerland. In total, 287 respondents who participated in organized sport before the age of 18 years completed a French version of the IViS (Vertommen et al., 2016). Even though less than 20% of the participants practiced their sport at the elite level, the prevalence rates observed were higher than those observed by Ohlert and colleagues: Among the respondents, almost 87% declared having suffered psychological violence, 38% physical violence, and 33% sexual violence. Almost all elite athletes who competed at the international level reported psychological violence (94%) and almost half the athletes who competed at the national level experienced physical (46%) and sexual violence (43%). Although individuals in positions of authority can be perpetrators of violence, it appears that the IV experienced by athletes primarily comes from their peers (teammates or opponents; Vertommen et al., 2016; Hauw et al., 2021). The higher the level of competition, the more coaches are identified as perpetrators of physical violence (Alexander et al., 2011; Hauw et al., 2021) and psychological violence (Stirling and Kerr, 2008). The same goes for sexual harassment: The higher the level of competition, the more the involvement of peers decreases while that of coaches increases (Alexander et al., 2011). There is currently little information about IV perpetrated by parents or others involved in sport (Parent and Fortier, 2017). However, the scandal surrounding Larry Nassar indicates that medical staff can also abuse their power relationship to mistreat athletes (Mountjoy, 2019), highlighting the need to consider all those involved in the athlete’s sport environment (e.g., medical staff and parents) in research on IV in sport. These three retrospective studies drew a portrait of the IV experiences in organized and elite sport measured retrospectively. Therefore, they do not allow us to precisely assess the timing and duration of the violence experience in athletes’ careers.

From this perspective, assessing the experiences of IV reported by athletes still practicing an organized sport at the time of the study was relevant. In the United Kingdom, Alexander et al. (2011) recorded prevalence estimates of 75% for psychological violence, 24% for physical violence, and 3% for sexual violence in recreational athletes under the age of 16. The prevalence rates observed by Parent and Vaillancourt-Morel (2020) in Canada were even higher: Among the 1,055 athletes aged between 14 and 17 years who completed The Violence Toward Athletes Questionnaire (VTAQ, Parent et al., 2019), 79% reported at least one experience of psychological violence followed by 39% reporting physical violence, 35% reporting neglect, and 28% sexual violence. The results of these last three studies revealed the relevance of questioning children and adolescent athletes and seemed to identify critical periods in the picture of current prevalence of sport-related IV. Young athletes’ perceptions of IV also enriched the perspectives for prevention and management.

Sport, in addition to school and family, is an important context in which children’s and adolescents’ experiences can be greatly influenced by the adults who take care of them (e.g., parents, teachers, and coaches) and by other children/adolescents (e.g., peers). Recent studies conducted in the family and school contexts have also demonstrated the relevance of conducting research with children and adolescents to measure parental neglect (Vanderminden et al., 2019), corporal punishment (Finkelhor et al., 2019) as well as teen dating violence (Beckmann et al., 2019). Also, as stated by Vertommen and Parent (2020), “including children’s voices and experiences in IV in sport research would be a welcome and valuable innovation” (p. 389). However, focusing on children and adolescents in research raises several issues in the study design. One of the key issues among them is the data reliability that is strongly grounded on the awareness of the participants of having experienced violence, which could depend on culture and on how the questions are asked (Fraga, 2016). Fortier et al. (2020a) stated that violence in sport is a concept that young athletes misunderstand or do not address at all. Sport culture could be also a strong cultural pressure to accept violence as natural (Young, 2019) and bring athletes to underreport it. Thus, study of IV experienced by young athletes requires adapting the questions in order to ensure their understanding and preserve the sensitivity of the young participants. According to Ybarra et al. (2009), questionnaires on sensitive topics should be taken with young people to provide accurate estimates of prevalence and avoid floor effects while ensuring that the impact of these questions on young people is understood.

The VTAQ (Parent et al., 2019) is the only validated questionnaire that assesses all forms of IV toward children and adolescents in sport perpetrated by coaches, parents, and peer athletes. Despite its validity, this questionnaire presents several limitations. First, the length of the questionnaire (73 items) may favor the appearance of a burden effect: The participants’ responses (and therefore the data) may be less precise as they progress through the questionnaire (e.g., Galesic and Bosnjak, 2009; Rolstad et al., 2011). Reliable but short or very brief versions of questionnaires are therefore key to responding to the growing need for rapid assessment of psychological states or for monitoring dynamics of change in various situation (e.g., Salisbury et al., 2005). In addition, a certain heaviness in thinking about unpleasant experiences can accumulate and result in less precise data, further from the respondents’ reality. Second, responding with frequencies can be more difficult than giving yes/no answers, especially for adolescents. Responding clearly and consistently may be more challenging for them, given that, as stated by Smith and Platt (2013) for young respondents, “ambiguities are enhanced, satisficing is more likely, and responses are more sensitive to cognitive burden” (p. 17). Third, the absence of a do not know response and the large number of questions that young participants must answer about a sensitive subject can build to a certain “uneasiness” as the questionnaire is completed, thus potentially influencing the quality of the data. Also, Raaijmakers et al. (2000) found that younger adolescents give more do not know responses (as age increases, the number decreases); therefore, the absence of this option could result in either a missing value or a random answer, both lowering the quality of the data. Finally, the way the VTAQ was created may lead us to miss violence perpetrated by others (e.g., medical staff members, club, or federation leader). Another point that should be added is related to the complexity of precisely reflecting the experiences of violence over the entire sport carrier. Krosnick and Presser (2010, p. 288) stated that the retrieval of information encoded in memory is affected by “both the information’s salience and the elapsed time since the information was coded”; therefore, they encouraged defining a short and recent reference period (in our context, this would be last year and not the entire career period). The IViS (Vertommen et al., 2016) is a shorter questionnaire than the VTAQ, and it aims to measure the prevalence of the three main forms of VI and neglect. However, this questionnaire measures the phenomenon retrospectively with questions on frequency (once, a few times, and regularly/often), and it asks adults about their past experiences in sport. It is therefore not suitable for a young audience and does not measure the current experience of IV.

To summarize, IV experienced by young athletes is a growing concern in sport psychology. The data obtained in Europe and North America reveal the existence of psychological, physical, and sexual violence that needs to be measured with athletes currently practicing their sport. The current tools for measuring these three forms of violence are few in number and have limitations that we propose to address in this study. More precisely, we propose to develop a short tool for measuring IV in sport adapted to a young audience. The participants can answer yes/no/I do not know, which should facilitate their reflections and therefore their answers to the questionnaire. Moreover, all potential perpetrators of IV can be cited.

In Switzerland, in 2014, the federal sports office OFSPO reported that more than 80% of Swiss children between the ages of 10 and 14 practiced 3 h or more of physical activity per week and 70% of Swiss adolescents from 15 to 19 engaged in between 3 and 10 h of physical activity per week. Despite the large number of Swiss children and adolescents participating in sport and physical activity and despite the cases of violence recently unveiled in the press by Swiss athletes,^1^ only one study has sought to draw a portrait of the IV experienced by young Swiss athletes retrospectively (see Hauw et al., 2021). Thus, following our first prevalence study based on retrospective design, our aim was to assess the prevalence of IV in sports in French part of Switzerland given voices directly to young athletes. Facing the important methodological limitations of the instruments used for assessing the prevalence of IV in sport lived by young athletes, we also examine how young athletes used and understood our questionnaire.

## Materials and Methods

### Construction of the Questionnaire

The first section of our questionnaire assesses demographic and sport-related characteristics: respondents’ gender, year of birth and sport experience (including the organized sport practiced at the time of the study from a list of 43 of the most popular sports in Switzerland), number of practice hours per week, and level of competition. The second section assesses the experience of IV in the sport context and was mainly based on the French version of the IViS (Vertommen et al., 2016) previously translated into French (see Hauw et al., 2021). This section is divided into three subsections: (i) psychological violence, (ii) physical violence, and (iii) sexual violence. The psychological violence section encompasses 11 questions about aggressive verbal bullying, exaggerated negative comments about performance or body, threats, and neglect. The physical violence section is composed of seven questions on physical abuse and overtraining. The sexual violence section encompasses 14 questions on sexual violence, covering both harassment and sexual abuse.

While the study by Hauw et al. found that the translation of the questionnaire was suitable for an adult population, some of the sentences needed to be adapted for a young, targeted population and they have thus been rephrased. Indeed, based on the findings of McCarthy et al. (2010), we adapted the questions to take into account young athletes’ ages and developmental level and, therefore, their understanding of IV. Thus, the first level of adaptation of the French version of the IViS (Vertommen et al., 2016) was based on the Childhood Experiences of Violence Questionnaire (Walsh et al., 2008). More precisely, we adapted question wording to a young audience (e.g., “Has anyone ever said inappropriate things about you (for example, jokes about your body or your gender (girl/boy) that bothered you)?” instead of “You were the subject/victim of sexual remarks about your body and looks.”). Also, we added a final question to learn about how the participants perceived the questions (deeply disturbing or shocking) using a Likert-scale varying from 1: questions very shocking to 7: not shocking at all.

The second level of adaptation of the French version of the IViS (Vertommen et al., 2016) was based on the Yes/No/Do not Know scale of the Halpérin et al. (1996) questionnaire on child sexual abuse in order to simplify the respondents’ reflections and answers. Moreover, given that the questionnaire was addressed to a young audience, we used the organization of Halpérin et al. (1996) concerning harassment and sexual abuse (a first level of questioning on experiences of harassment followed by a second level of questioning on experiences of actual sexual abuse). This questioning thus appears to be progressive in terms of the seriousness of sexual experiences.

Finally, we adapted each subsection to end with the same two multiple choice answer questions: (i) Who was the perpetrator of the act of violence (i.e., another athlete, a coach, and an institutional leader)? and (ii) Did you speak to someone about this violent experience (Yes/No)?

In a two-phase pre-test procedure, five young athletes (*M*: 15.4 years old) were first asked to assess the adequacy, clarity, and readability of the content during an open discussion. As a result, two items were adjusted during a team discussion under the supervision of the researcher responsible for the project because some respondents reported that these items were difficult to understand. Indeed, the item “Have you ever been yelled at during practice or competition?” was reframed as “Have you ever been scolded or yelled at during practice or competition?” and the item “Have you ever been ignored by someone during practice or competition, and it hurt you?” was reframed as “Have you ever been ignored by someone during practice or competition and that made you sad?” Second, three other young athletes aged 14 and 15 years assessed and confirmed the adequacy, clarity, and readability of the questionnaire content.

### Online Format

We developed and administered an online-format questionnaire for two main reasons. First, the vast majority of Europeans between 16 and 24 years old has access to the Internet and thus, recruitment was facilitated (see Frippiat and Marquis, 2010). Second, online questionnaires are described as being less intrusive, allowing participants to report more unusual or socially undesirable behavior (Frippiat and Marquis, 2010).

### Data Collection

After the study received the approval of the Ethics Committee of the University of Lausanne (E_SSP_112019_00001), participants were recruited on a voluntary basis through several recruitment strategies, such as the distribution of a hyperlink to the study *via* mailing lists of sport partners, the distribution of flyers to sport associations and competition venues (we asked both to redirect the link to participants through their own networks), and advertising of the study *via* social media in the French-speaking part of Switzerland. Data collection took place between December 2019 and April 2020, and the first step was sending the following: a presentation of the study, the questionnaire, and the inclusion criterion of the population (youth athletes between 14^2^ and 18 years old). By clicking on the link, the participants were informed about the content of the survey and were then redirected by a link to the online questionnaire. We also asked the participants to promote our study on their own social networks to allow us to reach a greater number of athletes through the technique of snowball sampling.

The distribution of the computerized questionnaire to the target population was carried out using eSurvey software. Respondents could only continue the study after accepting the informed consent request. They had the option to stop or end the survey at any time or any stage.

### Participants

A total of 329 young athletes began to respond to the questionnaire but 119 stopped after the sociodemographic section and did not start the section related to IV. Therefore, the sample size of this study was 210 young French-speaking athletes.

Among those who started the IV section, all answered the questions on physical violence (*n* = 210), 182 respondents (87%) answered on psychological violence, 165 on sexual violence (79%), and 155 on questions in the section on sexual abuse (72%). The two parts of the sexual violence subsection (i.e., sexual harassment and sexual abuse) did not show much difference in terms of response rate. There was no significant relationship between the indicator of response on the psychological violence questionnaire (respectively, sexual violence and sexual abuse) and gender and age group (*p* < 0.01), nor with the indicator of presence/absence of the violence measured just before in the questionnaire (*p* < 0.01).

To sum up, we noted that the number of respondents slightly decreased as the IV sections progressed, but with the study design we could not discriminate between the various possible reasons, such as interview-fatigue or non-response due to a troubling issue.

### Statistical Analyses

In order to estimate the prevalence of IV in sport among young people, the percentage was estimated with a confidence interval (CI) of 99%. Then, gender differences were tested with an independent samples t-test for continuous variables or the *χ*^2^ test of independence for categorical variables. To determine the relationship between the subgroups and the occurrence or not of psychological, physical, and sexual violence, we performed a *χ*^2^ test of independence. Descriptive statistics were calculated to analyze respondent-questionnaire interactions [percentage of do not know response (DKRs), per item and per subsection, and the item on the disturb/shock impact of the items] and to describe the sample in terms of demographics and sport variables.

For the items on the perpetrators of violence, which allowed multiple choice, we reported the cumulated percentages, i.e., either alone or with other perpetrators.

The level of statistical significance was set at 0.05. All statistical analyses were performed using Jamovi software version 1.6.23.0.

## Results

### Sociodemographic and Sport Characteristics of Respondents

The characteristics of the respondents are summarized in Table 1. The respondents were between 14 and 18 years old (*M*: 16.51, *SD*: 1.16) with 56% young female athletes. The sports practiced by the respondents were mostly team sports and individual sports (respectively, 32 and 30%) and about a third practiced their sport at the national competitive level (35%) and more than half of them (50%) practiced their sport 3 to 6 h per week.

**Table 1:** Sociodemographic and sport characteristics of the study sample and distribution by gender (*χ*^2^ or *t*-test results).

		Total sample	Male	Female	Statistic
		(*N* = 210)	(*N* = 92)	(*N* = 117)	(*p*)
		*N* (%)	*N* (%)	*N* (%)	
		*M* (*SD*)	*M* (*SD*)	*M* (*SD*)	
Age					7.89 (0.096)
		16.51 (1.16)	16.71 (1.06)	16.35 (1.21)	
Secondary school					4.93 (0.026)
	Yes	52 (24.8)	16 (17.4)	36 (30.8)	
	No	158 (75.2)	76 (82.6)	81 (69.2)	
Type of sport participation^1^					18.8 (<0.001)
	Individual sports	64 (30.5)	35 (38.0)	29 (24.8)	
	Team sports	67 (31.9)	15 (16.3)	52 (44.4)	
	Opposition sports	48 (22.9)	26 (28.3)	21 (17.9)	
	Outdoor sports	31 (14.8)	16 (17.4)	15 (12.8)	
	Sport practice					1.35 (0.929)
0-2 h/week		22 (10.5)	10 (10.9)	12 (10.3)	
3-4 h/week		49 (23.3)	21 (22.8)	28 (23.9)	
5-6 h/week		57 (27.1)	25 (27.2)	32 (27.4)	
7-8 h/week		36 (17.1)	16 (17.4)	20 (17.1)	
9-10 h/week		20 (9.5)	7 (7.6)	13 (11.1)	
>10 h/week		26 (12.4)	13 (14.1)	12 (10.3)	
Sport level					4.49 (0.343)
	International	18 (8.6)	11 (12.0)	6 (5.1)	
	National	73 (34.8)	33 (35.9)	40 (34.2)	
	Cantonal^2^	47 (22.4)	19 (20.7)	28 (23.9)	
	Regional	27 (12.9)	9 (9.8)	18 (15.4)	
	Leisure	45 (21.4)	20 (21.7)	25 (21.4)	

1 We classified the type of sport in four main categories: individual sports (technical artistic sport), team sport (all collective sport), dual sport (individual sport where there is a direct confrontation with the opponent, such as racquet sport, and combat sport), and outdoor sport (e.g., kayaking and mountain biking).

2 In Swiss sport, the cantonal level corresponds to the North American provincial level.

### Reported Prevalence of IV in the Overall Sample

Regarding the percentage of respondents who experienced at least one event of IV, 75% declared psychological violence (*n* = 137 of 182 respondents, 99% CI 66.1–83.0%), 53% physical violence (*n* = 111 of 210 respondents, 99% CI 43.8–61.8%), 28% sexual violence (*n* = 46 of 165 respondents, 99% CI 19.3–37.7%), and 21% no violence.

### Reported Prevalence of IV According to Sociodemographic and Sport Characteristics

The prevalence of IV is shown in Table 2 in terms of sociodemographic and sport characteristics. The results indicate that male respondents reported more exposures to an incident of physical violence than females (64.1% vs. 43.6%; *p* = 0.003), whereas female respondents reported more exposures to psychological violence than males (81% vs. 67.1%; *p* = 0.033). Regarding the type of sport practiced, there was a significant difference in the reports of psychological and physical violence - with young respondents practicing individual sports reporting significantly more physical or psychological violence - but not sexual violence (*p* = 0.022; *p* < 0.001; *p* = 0.488). There was no significant difference in prevalence or level-of-competition groups for the three forms of IV. There was a significant difference in the mean age of those who experienced and those who did not experience physical violence (*p* = 0.004; *M*: 16.73 for those who reported experience against 16.27), but not psychological violence (*p* = 0.537) or sexual violence (*p* = 0.312).

**Table 2:** Reported prevalence of the three forms of sport-related IV per subgroup (low threshold measure, i.e., at least one experience; number and percent among subgroup with *χ*^2^ results for distribution among subgroups).

		Psychological violence		Physical violence		Sexual violence	
		*N* = 137	*χ* ^2^	*N* = 111	*χ* ^2^	*N* = 46	*χ* ^2^
		*N* (%)	(*p*)	*N* (%)	(*p*)	*N* (%)	(*p*)
Gender			4.53		8.72		6.70
			(0.033)		(0.003)		(0.010)
	Female	85 (81.0)		51 (43.6)		34 (35.8)	
	Male	51 (67.1)		59 (64.1)		12 (17.4)	
Type of sport participation			9.67		21.11		2.43
			(0.022)		(<0.001)		(0.488)
	Individual sports	40 (85.1)		46 (71.9)		14 (30.4)	
	Team sports	50 (78.1)		22 (32.8)		14 (28.9)	
	Opposition sports	25 (58.1)		24 (50.0)		8 (19.5)	
	Outdoor sports	22 (78.6)		19 (61.3)		10 (35.7)	
Sport level			7.95		6.35		4.64
			(0.093)		(0.175)		(0.347)
	International	15 (88.2)		14 (77.8)		5 (31.1)	
	National	51 (76.1)		33 (45.2)		11 (18.3)	
	Cantonal	31 (86.1)		25 (53.2)		11 (35.5)	
	Regional	14 (60.9)		14 (51.9)		7 (35.0)	
	Leisure	26 (66.7)		25 (55.6)		12 (31.6)	

### Perpetrator of the Act of Violence

Most of the identified perpetrators of violence were peers (respectively, 55.5%, 57.7, and 55.2%, Table 3), either alone or with other perpetrators. Coaches were the second most cited perpetrators and more frequently mentioned as the perpetrators of psychological violence (41.6%) compared to physical or sexual violence (respectively, 27.9 and 34.8%), either alone or with other perpetrators. Conversely, institutional leaders made up a small part of the IV perpetrators.

**Table 3:** Perpetrator profiles for the three forms of IV.

Perpetrator profiles	Psychological violence	Physical violence	Sexual violence
	(*N* = 137)	(*N* = 111)	(*N* = 46)
	*N* (%)	*N* (%)	*N* (%)
Peer athlete	76 (55.5%)	64 (57.7%)	24 (52.2%)
Coach	57 (41.6%)	31 (27.9%)	16 (34.8%)
Leader	8 (5.8%)	4 (3.6%)	3 (6.5%)

The sum of percentages can be higher than 100% as a respondent may have suffered violence by multiple perpetrators.

### Speaking Out According to the Type of IV

Almost half the participants who indicated experiencing psychological violence had reported it to someone (46.7%), whereas 38.7% of those who experienced physical violence had reported it, and 28.3% of those who experienced sexual violence had reported it.

### Sexual Harassment and Sexual Abuse

Among the 155 respondents who completed this subsection, 36 reported sexual harassment (23.8%). There was no significant difference in the proportions for gender (*p* = 0.389), type of sport (*p* = 0.658), or level of competition (*p* = 0.944). When we compared the results for the two types of sexual violence, we found broad concordance: 75% gave the same answer, that is 14% (*n* = 21) experienced both types of sexual violence, and 61% (*n* = 92) did not experience either of these forms of sexual violence. However, 25% indicated different responses: 15% indicated sexual abuse, but no sexual harassment (*n* = 23) and 10% indicated sexual harassment but no sexual abuse (*n* = 15). This result underlines the importance of dissociating these two forms of sexual violence in the construction of research instruments (questionnaires and interview guides).

### The Use of the Questionnaire by Participants

#### Do Not Know Response

Among the items related to physical violence, item 2 elicited the fewest DKRs (“slapped or punched,” 1 respondent: 0.5%) and item 6 the most DKRs (“violently grabbed,” 12 respondents: 5.7%). For the whole physical violence subsection, respondents had between 0 and 4 DKRs and an average of 0.22 DKRs for each of the 10 items (*SD*: 0.56). Of the items related to psychological violence, item 10 elicited the fewest DKRs (“forced to take drugs,” 0 respondent) and item 1 the most DKRs (“intimidated,” 17 respondents: 9.3%). For the whole psychological violence subsection, respondents had between 0 and 5 DKRs and an average of 0.57 DKRs for each of the 14 items (*SD*: 1.00). Among the items related to sexual violence (part A – Sexual harassment), item 1 elicited the fewest DKRs (“embarrassed to be alone,” 1 respondent: 0.6%) and item 2 the most DKRs (“a look causing discomfort,” 9 respondents: 5.5%). For this part of the sexual violence subsection, respondents had between 0 and 3 DKRs and an average of 0.16 DKRs for each of the 6 items (*SD*: 0.51). Among the items related to sexual violence (part B – Sexual abuse), item 9 elicited the fewest DKRs (“showed his/her private parts,” 0 respondent) and item 10 the most DKRs (“looking at private parts without consent,” 9 respondents: 5.5%). For this part of the sexual violence subsection, respondents had between 0 and 2 DKRs and an average of 0.13 DKRs for each of the 8 items (*SD*: 0.37).

#### Perception of “Shocking or Deeply Disturbing” Questions

Violence in sport can be a delicate subject to tackle, and the final question of the questionnaire aimed to assess how shocking or deeply disturbing the questions were perceived to be (low scores meaning shocking). On average, participants found the questions relatively not shocking/deeply disturbing (*M*: 4.85, higher than 4, the midpoint of the scale) but with some heterogeneity in their answers (*SD*: 2.10).

## Discussion

The objectives of this study were to (i) assess the prevalence of violence in youth sport in the French-speaking part of Switzerland and (ii) examine how participants used and understood the questionnaire design. The discussion is divided into three parts: we first examine the results in relation to other prevalence studies, we then discuss the strengths and limitations of our instrument, and last, we discuss the IV reporting design.

### Prevalence Rate Studies: Methodological Specificities

In the present study, psychological violence was the most reported by the young athletes, which corresponds to the results of recent studies (Peltola and Kivijärvi, 2017; Ohlert et al., 2020; Parent and Vaillancourt-Morel, 2020; Hauw et al., 2021). Also, in line with previous studies (Alexander et al., 2011; Hauw et al., 2021), our results highlighted that the perpetrators of psychological and sexual violence in the sport context were mainly peers. Conversely, Kerr et al. (2019) observed that most psychological violence was experienced from coaches, followed by peers. Regarding the French-speaking Swiss prevalence rates, Hauw et al. (2021) found reports of 87% psychological, 37.7% physical, and 32.8% sexual violence among former athletes, whereas we observed that 75.3% of our participants declared psychological violence, 52.9% physical violence, and 27.9% sexual violence. Similar results were observed by Kerr et al. (2019), who noted that higher percentages of retired athletes reported experiencing at least one behavior across all forms of IV compared with current athletes. Thus, our data reinforce the idea that psychological violence is the form of IV that is most reported by athletes, whether in present or past sport experiences.

Despite the apparent similarities and differences between previous studies and the present study, the interpretation and comparison of prevalence rates must be made with caution and need to be discussed. Three points are relevant here. First, the conceptions of IV that researchers use vary, and therefore, their way of asking and organizing questions in each questionnaire will likewise vary. Although Peltola and Kivijärvi (2017, p. 958) considered experiences of violence “[…] as broadly as possible, including both acts defined as crimes by the law and acts with more ambiguous legal status, such as bullying, harassment, emotional abuse and corporal punishment”, Vertommen et al. (2016), Hauw et al. (2021), Ohlert et al. (2020), and the present authors adopted the definition of violence as documented in article 19 of the Convention on the Rights of the Child (United Nations, 1989): “[…] all forms of physical or mental violence, injury and abuse, neglect or negligent treatment, maltreatment or exploitation, including sexual abuse while in the care of parent(s), legal guardian(s) or any other person who has the care of the child.” Based on the WHO typology of violence to determine the four forms of IV (i.e., sexual, psychological, physical violence, and neglect; Krug, 2002), Parent and Vaillancourt-Morel (2020) used the VTAQ, which is based on the WHO definition of violence: “the intentional use of physical force or power, threatened or actual, against oneself, another person, or against a group or community, that either results in or has a high likelihood of resulting in injury, death, psychological harm, mal-development or deprivation” (Krug, 2002, p. 5). Similarly, Kerr et al. (2019) considered questions about harm, maltreatment, and neglect. In addition, the VTAQ (Parent et al., 2019) considers physical violence to be centered on the nature of the gestures (physical contact), whereas the IViS (Vertommen et al., 2016) considers physical violence to be centered on the consequences of the act (with and without physical contact). Thus, data categorized as physical violence in the present and other results (Vertommen et al., 2016; Ohlert et al., 2020; Hauw et al., 2021) are presented as psychological violence in other studies (Parent and Vaillancourt-Morel, 2020). Thus, our results are to some extent comparable to those obtained by Hauw et al. and Vertommen et al., but these data were obtained retrospectively (and therefore are ultimately not very comparable). As proposed by Fortier et al. (2020b), considering the nature of the act in organizing the forms of physical and psychological violence would allow us to compare prevalence rates.

Second, prevalence rates depend on the type of frequency scale used. While the present study poses Yes/No/Do not Know questions, the IViS (Vertommen et al., 2016) uses a Once, A few times, and Regularly/Often scale. Peltola and Kivijärvi (2017) asked Never, Sometimes, or Often questions, and Kerr et al. (2019) mixed frequency scales and Yes/No questions (i.e., choices of “Yes,” “No,” “Often,” and “Occasionally”). The VTAQ (Parent et al., 2019) specifies 0: Never, 1: Rarely, one to two times, 2: Sometimes, three to 10 times, and 3: Often, more than 10 times, but Parent and Vaillancourt-Morel coded 0: no violence and 1: at least one event of at least one type of violence to report their results. On this point, the Yes/No/Do not know scale used in the present study may have simplified the respondents’ reflections and answers about their current experience of IV in sport. Future studies should address this methodological decision as it may influence respondents’ answers and therefore the data obtained regarding sport-related IV.

Third, the participants interviewed in the different studies did not have the same characteristics. The prevalence rates concerned, as in the present study, current athletes (Peltola and Kivijärvi, 2017; Parent and Vaillancourt-Morel, 2020), current and former athletes (Kerr et al., 2019), or former athletes (Vertommen et al., 2016; Ohlert et al., 2020; Hauw et al., 2021). Moreover, these prevalence rates accounted for experiences at varying times without mentioning the duration. Various performance and practice levels within each study can also be observed, and in each sample, very different types of sport were included and analyzed. For example, whereas Hauw et al. (2021) considered four main types of participation (i.e., individual, team, outdoor, and opposition), Parent and Vaillancourt-Morel (2020) considered only individual and team sports. This confirms the methodological need to standardize measurement instruments. It also underlines the need to define the conceptual and theoretical components that underlie the construction of study designs. Based on our results and their possible comparison with previous studies, it is important that researchers interpret their data according to the type of measurement they use (retrospective or direct), the type of sport, and the level of the participants’ practice. Thus, future studies should help situate the various sports in relation to each other in terms of the IV prevalence rate. For example, is peer violence reported more often in team sports? Is coach abuse reported more often in early specialization sports?

### Strengths and Limitations of our Instrument

The results of the present study suggest that the questionnaire we developed was usable for a young audience because (i) there was only a slight decrease in respondent replies as the IV section progressed, (ii) the DKRs were low, and (iii) participants mainly found the questions relatively not shocking/disturbing. In addition, our study brings new data that aim to draw a global portrait of the sport-related IV phenomenon from young athletes’ points of view. More precisely, our results suggest that young Swiss athletes under 18 years not only understand the meaning of violent behavior from coaches or peers but are also able to describe how they experience it (e.g., have been shocked, disturbed, or not). This result also suggests the potential value of further and in-depth studies to learn more about the experience of young athletes.

Analysis of respondent-questionnaire interactions is a good indicator of the usability of the questionnaire, but some limitations must be discussed. First, the use of the Yes/No/Do not Know scale seemed easier for the respondents than a frequency scale. This easiness for the respondent nevertheless has a counterpart for the researcher, who loses the possibility to analyze frequencies (see the IViS of Vertommen et al., which can assess the severity of the IV using a frequency scale). Second, each indicator used in our items captures a precise picture of the phenomenon and therefore invites researchers to be careful in formulating these indicators. Thus, having undergone a form of IV once (i.e., hearing inappropriate things about oneself one time) cannot be compared to continuous solicitations (i.e., sexual harassment) nor to the experience of having been the victim of a severe form of abuse (e.g., unwanted penetration). Third, regarding the organization of sexual violence into two parts (i.e., harassment and sexual abuse) accounted for the progression in the seriousness of sexual experiences. However, this might have affected the clarity of the data with the multiplication of indicators ultimately giving a blurry picture of the sexual violence experienced by the participants. Fourth, asking participants about perpetrators with a closed question limits responses about other potential perpetrators, such as parents or medical staff, who are important actors in the careers of young athletes’, especially in early specialization sports (e.g., gymnastics, diving, and soccer). Finally, we are unable to explain why a good number of participants did not complete the questionnaire, as it was quite short and easy to complete. Future studies should assess the reasons participants may find the questionnaire difficult to answer, especially across generations (e.g., it could be easier for the youngest athletes to answer a questionnaire on the phone without being particularly distracted by a notification).

To resume, more studies are needed to compare results and thus draw a finer-grained picture of IV in sport and more importantly, the data collection instruments need to be harmonized. Depending on the type of study and its objectives, this harmonization might be multifactorial: A first approach (Yes/No scale) followed by greater detail on the phenomenon/experiences of violence (use of frequency scales). Finally, the types of measurement would benefit from being clearly specified when researchers build their study designs.

### IV Reporting Design: Toward Time-Sensitive IV Research

The objective of measuring the prevalence of IV against children in sport has prompted researchers to reflect on the types of reporting (Vertommen and Parent, 2020). Beyond the so-called “retrospective” or “direct” aspect of measurement, there is a wide range of information that could be useful for discriminating and comparing reports of IV but that would need to be specified in the types of reporting. For example, are the reports from (i) adults reporting their past IV experiences in childhood or adolescence, with the IV experiences interrupted or ended?; (ii) adults reporting past IV experiences that continue today?; (iii) children or adolescents reporting past IV experiences that have been interrupted or ended?; (iv) or are they continuing today?; and a critical question for all forms would be: (v) have the past and/or current IV experiences lasted a long time? Thus, beyond the usual limitations of retrospective measures (see Moody et al., 2018), the notion of time should receive very careful attention. First, the time between the experience of violence and the present moment might be more or less long, but there is little chance that it will be very short (i.e., it is unlikely that the athlete completes the questionnaire immediately after having experienced sport-related violence). Second, the experience of violence may have lasted for a longer or shorter time (e.g., an isolated and occasional act of physical violence from the coach vs. psychological harassment that has lasted for years). Rather than focusing only on the direct or retrospective aspect of a research design (which is ultimately difficult to do since it is difficult to determine at what point a measurement is strictly retrospective), researchers should therefore also consider the various dimensions of the variable “time” of IVs. Indeed, we suggest that study designs for examining sport-related IV should consider IV in terms of (i) the age of the participants, (ii) the moment in the career that is addressed by the questions (and therefore the time elapsed between the experiences of IV and the response to the questionnaire), and (iii) the duration of the experience of violence.

This type of taxonomy of research designs would allow researchers to qualify/nuance the results depending on when the IV experiences occurred, how long they lasted, and whether or not they were over when the questionnaire was answered.

## Conclusion

Measuring the prevalence of IV in the sport context provides important information that allows researchers and sport stakeholders to understand more fully the negative experiences of their young athletes in several countries. However, the measurements taken since the first prevalence study (Alexander et al., 2011) have been difficult to interpret and compare for several reasons. For a phenomenon as complex as IV, it is difficult to develop a single instrument that covers everything, and mixed methods designs may offer a solution in the future, especially if they include interviews conducted by confirmed sport psychologists. In addition, a progression in the strength of research designs should also be considered in future studies: While certain methodologies can be used to obtain a preliminary inventory (e.g., prevalence studies), others will be useful for exploring IV experiences with more finesse (e.g., reconstruction of traces of past activity). Overall, a key point here seems to reside in strengthening the relationship between seeking information on prevalence and conducting research aimed at having an effect on interventions; in other words: scientific rigor in relation to the relevance of the intervention (Schon, 1984).

## Data Availability Statement

The original contributions presented in the study are included in the article/supplementary material, further inquiries can be directed to the corresponding author.

## Ethics Statement

The studies involving human participants were reviewed and approved by the Ethics Committee of the University of Lausanne (E_SSP_112019_00001). Written informed consent to participate in this study was provided by the participants’ legal guardian/next of kin.

## Author Contributions

All authors listed have made a substantial, direct and intellectual contribution to the work, and approved it for publication.

## Conflict of Interest

The authors declare that the research was conducted in the absence of any commercial or financial relationships that could be construed as a potential conflict of interest.

## Publisher’s Note

All claims expressed in this article are solely those of the authors and do not necessarily represent those of their affiliated organizations, or those of the publisher, the editors and the reviewers. Any product that may be evaluated in this article, or claim that may be made by its manufacturer, is not guaranteed or endorsed by the publisher.

## Footnotes

^1^ https://www.rts.ch/play/tv/temps-present/video/violences-sexuelles-un-sport-a-haut-risque?urn=urn:rts:video:11477813

^2^In Switzerland, participants older than 14 years can give consent to participate in a study without needing their parents’ consent.
